# Association between Surrounding Greenness and Mortality: An Ecological Study in Taiwan

**DOI:** 10.3390/ijerph17124525

**Published:** 2020-06-23

**Authors:** Hsiao-Yun Lee, Chih-Da Wu, Yi-Tsai Chang, Yinq-Rong Chern, Shih-Chun Candice Lung, Huey-Jen Su, Wen-Chi Pan

**Affiliations:** 1 Department of Leisure Industry and Health Promotion, National Taipei University of Nursing and Health Sciences, Taipei 112, Taiwan; hsiaoyun07@ntunhs.edu.tw; 2 Department of Geomatics, National Cheng Kung University, Tainan 701, Taiwan; chidawu@mail.ncku.edu.tw (C.-D.W.); cyt.nawo@gmail.com (Y.-T.C.); dcpast1208.rive@gmail.com (Y.-R.C.); 3 National Institute of Environmental Health Sciences, National Health Research Institutes, Miaoli 350, Taiwan; 4 Research Center for Environmental Changes, Academia Sinica, Taipei 115, Taiwan; sclung@rcec.sinica.edu.tw; 5 Department of Environmental and Occupational Health, National Cheng Kung University, Tainan 701, Taiwan; HJSU@mail.ncku.edu.tw; 6 Institute of Environmental and Occupational Health Sciences, National Yang-Ming University, Taipei 112, Taiwan

**Keywords:** greenness, mortality, Taiwan

## Abstract

Exposure to surrounding greenness is associated with reduced mortality in Caucasian populations. Little is known however about the relationship between green vegetation and the risk of death in Asian populations. Therefore, we opted to evaluate the association of greenness with mortality in Taiwan. Death information was retrieved from the Taiwan Death Certificate database between 2006 to 2014 (3287 days). Exposure to green vegetation was based on the normalized difference vegetation index (NDVI) collected by the Moderate Resolution Imagine Spectroradiometer (MODIS). A generalized additive mixed model was utilized to assess the association between NDVI exposure and mortality. A total of 1,173,773 deaths were identified from 2006 to 2014. We found one unit increment on NDVI was associated with a reduced mortality due to all-cause (risk ratio [RR] = 0.901; 95% confidence interval = 0.862–0.941), cardiovascular diseases (RR = 0.892; 95% CI = 0.817–0.975), respiratory diseases (RR = 0.721; 95% CI = 0.632–0.824), and lung cancer (RR = 0.871; 95% CI = 0.735–1.032). Using the green land cover as the alternative green index showed the protective relationship on all-cause mortality. Exposure to surrounding greenness was negatively associated with mortality in Taiwan. Further research is needed to uncover the underlying mechanism.

## 1. Introduction

Non-communicable chronic diseases result in a great health burden worldwide. According to statistics provided by the World Health Organization (WHO), cardiovascular-related and respiratory diseases significantly contributed to the disability-adjusted life years (DALY) [1,2]. Globally, ischemic heart disease ranks as the first (for males) and second (for females) in terms of global DALYs. Chronic Obstructive Pulmonary Disease (COPD), one of the major respiratory diseases, is the sixth leading causes of DALYs for both genders. Additionally, lung cancer contributes more than 40,000 DALYs and has the highest ranks among all types of neoplasm.

Earlier studies suggest the burden of major chronic diseases could be reduced by the surrounding greenness [3], and some evidence indicates the interplay between greenness and air pollution on mortality [4,5,6]. A study based on U.K. vital statistics found people exposed to higher levels of green space have lower mortality rates, and this positive relationship between income deprivation and all-cause mortality is attenuated by the green space [7]. A cross-sectional study in Australia demonstrated an inverse association between neighborhood greenness and cardiovascular diseases [8]. Recently, several studies based on large cohort profiles in Caucasian populations also found a negative relationship between greenness exposure and mortality. In 2016, James et al. found residential exposure to greenness is negatively associated with all-cause mortality in the Nurse Health Study [9]. Similar findings were observed in the U.S. [10], Switzerland [11], Canada [4,12], and Italy [13].

Despite the accumulative evidence in Caucasian populations, only limited studies have been conducted in Asian populations. A cohort study based on older Chinese participants found greater proximity to greenness is associated with a lower mortality risk [14], and an interaction between greenness and fine particulate matter (PM_2.5_) on mortality was observed [15]. Kim et al. found higher exposure to greenness is associated with decreased CVD mortality in the Korean metropolitan area [16].

However, few studies have utilized a nationally representative population to assess the greenness-mortality relationship in Asia. Therefore, we opted to evaluate the association of greenness exposure with mortality using a database of Taiwanese national vital statistics.

## 2. Materials and Methods

### 2.1. Cause of Death

Based on the International Classification of Disease, ninth/tenth revision, clinical modification codes ICD-9, ICD-10, cause of death information for the 2006–2014 period was retrieved from the Taiwan Death Certificate Database maintained by the Health and Welfare Data Science Center, Department of Health and Welfare (Taiwan). We specifically extracted information for cause of death, date of death, place of death, sex, and age of death. Total cause of death (except for death due to accident, suicide, or homicide) and deaths caused by respiratory diseases (ICD-9: 480–487,490–493; ICD-10: J10–18, J20–21, J40–47, J60–66, J68–69), cardiovascular diseases (ICD-9: 390–392, 393–398, 401–405, 410–414, 420–438, 440; ICD-10: I01–102.0, I05-I15, I20–25, I30–52, I60–71), and lung cancer (ICD-9: 162; ICD-10: C33–34) were used. We excluded death records with the place of death on isolated islands (e.g., Penghu Islets). All information was aggregated into a township level for the analyses. The Institutional Review Board of National Cheng Kung University approved the study protocol (NCKU HREC-E-106-228-2).

### 2.2. Index of Surrounding Greenness

**Figure 1:**
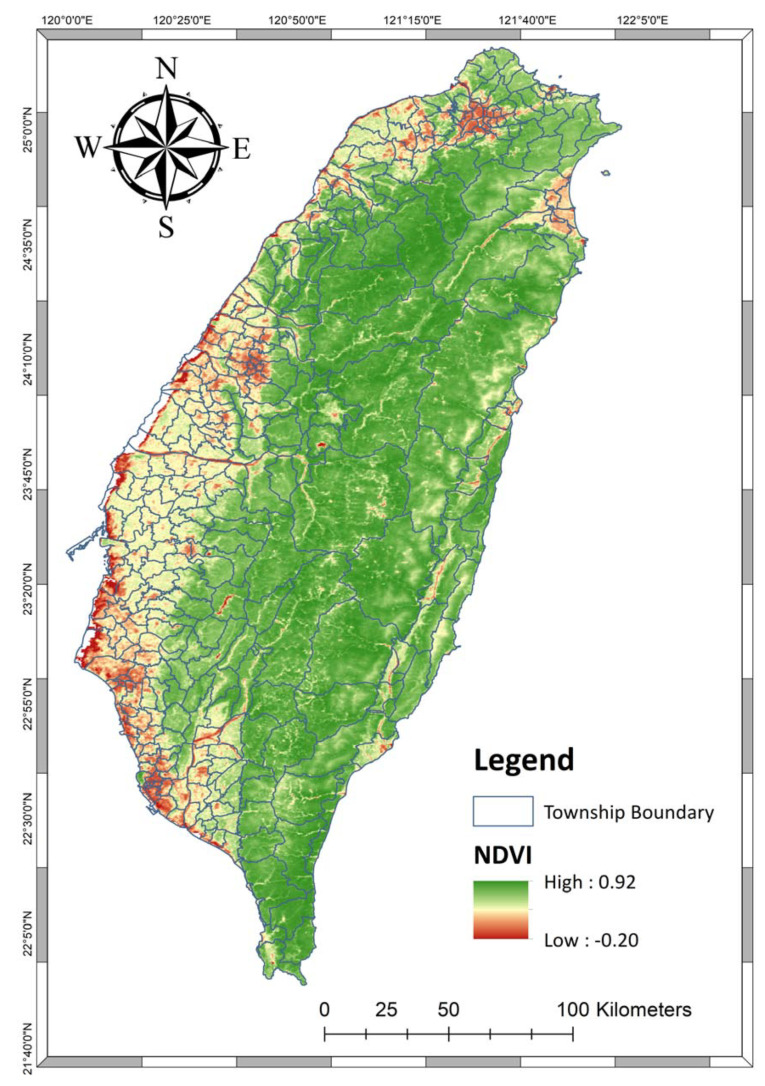
Spatial Distribution of NDVI from 2006 to 2014.

We primarily used the Normalized Difference Vegetation Index (NDVI) in 2006–2014 as the major index for greenness in this study. NDVI was calculated based on the information of near-infrared bands (NIR) as well as read bands (RED) measured by satellites, and it ranged from −1 to 1. A higher positive value indicates higher greenness coverage. NDVI information was extracted from the MOD13Q1 database (Version 5) which was collected by the US National Aeronautics and Space Administration (NASA) through the Moderate Resolution Imaging Spectroradiometer (MODIS) of the Terra satellite. MODIS provides NDVI images at a resolution of a 250-m grid every 16 days. Figure 1 shows the average NDVI in Taiwan between 2006 and 2014. A land-cover of forest and/or park was also used as the secondary greenness index in this study. We extracted the coverage of forests and parks from the Taiwan Land-use Investigation (2014) provided by the Taiwan Ministry of Interior. All greenness statistics were summarized in a township manner.

### 2.3. Information of Covariates

Information of annual township demographic (2006–2014) including age structure (0–14, 15–64, >64 years) and sex ratio (male/female) was provided by the Taiwan Department of Household Registration. We retrieved information of household annual income (2006–2014) from the Taiwan Economic Database maintained by the Ministry of Finance, Taiwan. We extracted the seasonal average of PM_2.5_ and NO_x_ between 2006 and 2014 from the Taiwan Air Quality Monitoring Network (TAQMN). A land-use regression (LUR) model developed previously was applied to model spatial and temporal distribution of PM_2.5_ [17]. Township levels of NO_x_ were interpolated by an ordinary kriging followed by a zonal statistics using ArcGIS 10.2 (ESRI, Redlands, CA, USA). The R-squared were 0.89 for PM_2.5_ and 0.29 for NO_x_. Daily temperature and precipitation was extracted from the Data Bank for Atmospheric Research covering the years 2006 to 2014. Information was aggregated in a seasonal fashion, and an ordinary kriging was applied to calculate the seasonal level of precipitation and temperature of each township.

### 2.4. Statistical Analysis

This study aimed to estimate the association between greenness and causes of death. NDVI was the primary greenness index and was analyzed in a continuous fashion. Percent of area for the forest, park, and forest/park in township levels was used as the secondary green index (continuous variable). This study had four outcome variables including total mortality, cardiovascular mortality, respiratory mortality, and lung cancer mortality.

Generalized additive mixed models (GAMM) in a Poisson setting were utilized to evaluate the greenness-mortality association. Models were adjusted for total township population (continuous), age (0–14, 15–64, >64 year), sex ratio (continuous), income (continuous), PM_2.5_ (continuous), NO_x_ (continuous), precipitation (continuous), temperature (continuous), and urbanization levels (high or low). Regression spline function with a proper degree of freedom (df) on precipitation (df = 3), temperature (df = 3), and seasonal trend (df = 10) was applied to control the confounding bias. To account for the temporal correlation of seasonal mortality in each township, we included random intercepts with a covariance structure of auto regressive 1 (AR1). To minimize the spatial autocorrelation, a cubic regression spline with 3 degrees of freedom on longitude/latitude coordinates was introduced. The longitude and latitude was based on the centroid of each township.

In addition, we performed stratified analyses to evaluate whether greenness-mortality association varies by demographical variables including sex ratio (< or ≥ median [107.3]), proportion of age at 15–64 years (< or ≥ median [71.9%]), annual household income (< or ≥ median [668,701 New Taiwan Dollar]), and area (urban or rural). We defined townships located in the six major cities (Taipei City, New Taipei City, Taoyuan City, Taichung City, Tainan City, and Kaohsiung City) as the urban area.

## 3. Results

Three causes of death (i.e., cardiovascular diseases, respiratory diseases, and lung cancer) and total cause of death between 2006 and 2014 are shown in Table 1. Totally, we identified 1,173,773 deaths in the study period (2006–2014). On the basis of the seasonal average, total cause of death was 96.09 per 100,000 people (standard deviation [SD] = 96.63). Number of deaths per 100,000 people caused by cardiovascular diseases, respiratory diseases, and lung cancer were 21.91 (SD = 21.76), 9.42 (SD = 9.87), and 5.89 (SD = 6.61), respectively. The average sex ratio (male/female) was 107.74% (SD = 8.11%), indicating a higher male population compared with females in Taiwan (2006–2014). In terms of age structure in township levels, age at 15–64 years was the dominant category (mean = 71.86%; SD = 2.97%). Note, the average seasonal level of NDVI was 0.51 (SD = 0.17), and 30% of the township area was covered by forest (mean area of forest = 30.07% [SD = 30.8]). The spatial distribution of average NDVI (2006–2014) was shown in Figure 1. Lower NDVI was found in the western and northern area where the major cities are located. Central and eastern Taiwan is primarily covered by mountains and forest, and therefore higher NDVI levels were found in these areas

In Table 2, we found a negative relationship between NDVI and all-cause mortality with statistical significance. With one unit increment on NDVI, the all-cause mortality was reduced by 9.9% (adjusted risk ratio [RR] = 0.901; 95% confidence interval [CI] = 0.862–0.941; *p*-value < 0.001) (Model 1), controlling age, sex ratio, annual household income, temperature, precipitation, and time trend. The adjusted RR of mortality due to cardiovascular diseases, respiratory diseases, and lung cancer were 0.892 (95% CI = 0.817–0.975; *p*-value = 0.012), 0.721 (95% CI = 0.632–0.842; *p*-value < 0.001), and 0.871 (95% CI = 0.735–1.032; *p*-value = 0.11) per one unit increase on NDVI, respectively. This negative NDVI-mortality association was consistent with further adjustment for NOx (Model 2) and urbanization levels (Model 3), and the statistical significance remained for all-cause, cardiovascular, and respiratory mortality. We found higher levels of NDVI were negatively associated with lung cancer mortality, although its statistical significance was marginal.

To assess whether the negative association of NDVI with mortality varied by population characteristics, we performed a stratified analyses based on sex ratio, age at 15–64, annual household income, and urbanization levels. In Table 3 the adjusted RR for NDVI exposure (one unit increment) and all-cause mortality were slightly stronger among townships with a lower sex ratio (0.877 [≥median] vs. 0.929 [<median]), higher age of 15–64 (0.876 [≥median] vs. 0.924 [<median]), higher annual household income (0.871 [≥median] vs 0.954 [<median]), and urban area (0.861 [urban] vs 0.923 [rural]), but none of them revealed a statistical interaction. However, NDVI was consistently associated with reduced all-cause, cardiovascular, respiratory, and lung cancer mortalities across different township characteristics.

We further performed sensitivity analyses whereby the green vegetation (i.e., NDVI) was replaced by green land coverage (e.g., area of forest) and found a statistically significant relationship between green land coverage and reduced mortality (Table 4). 

With a 10% increase in forest area, the adjusted RRs (95% CI) of mortality were 0.883 (0.866–0.900) (all-cause), 0.898 (0.880–0.917) (cardiovascular diseases), 0.903 (0.883–0.923) (respiratory diseases), and 0.884 (0.864–0.904) (lung cancer). Similar results were found for exposure to the forest/park area.

## 4. Discussion

This ecological study found higher levels of green vegetation (i.e., NDVI) was associated with a reduced all-cause and cause-specific (i.e., cardiovascular, respiratory, lung cancer) mortality in Taiwan, and this negative relationship was stronger for death due to respiratory diseases. The protective association of greenness on mortality was persistent across different population characteristics including age, sex ratio, annual household income, and urban/rural area. Findings based on green land coverage (e.g., forest coverage) consistently showed a negative relationship between greenness exposure and mortality.

Recent studies conducted in the Asian population support the association between greenness exposure and reduced mortality [14,15,16]. A longitudinal study based on an oldest-old Chinese population (Chinese Longitudinal Healthy Longevity Survey, CLHLS) found exposure to contemporaneous NDVI was negatively associated with all-cause mortality [15]. Participants with the highest quartile exposure to contemporaneous NDVI (250 m buffer) showed a 27% reduction in all-cause mortality compared to the reference group (i.e., first quartile). Change in contemporaneous NDVI buffer to 1250 m showed a similar result, and a linear trend was found for the NDVI-mortality relationship. A follow-up study based on this cohort profile (i.e., CLHLS) also uncovered an interaction between air pollution and surrounding greenness [15]. Ji et al. found contemporaneous NDVI significantly modified the association between 3-year average PM_2.5_ exposure and mortality. The PM_2.5_-mortality relationship tended to be positively linear for participants exposed to the first and second tertiles of contemporaneous NDVI. However, a non-linear relationship was found among those with the highest NDVI exposure (i.e., the third tertile) and a reversed U-shape was suggested. Another study conducted in a Korean population resided in metropolitan cities showed a negative association between NDVI exposure and mortality [16]. Kim et al. found non-accidental mortality was reduced by 0.59% with an interquartile range (IQR) increment of NDVI at an annual level. A similar trend was found for deaths due to cardiovascular diseases, but opposites relationship was found for respiratory and lung cancer mortality. On the contrary, our findings showed some different pattern compared to other studies conducted in Asian populations. We observed a negative association between NDVI exposure and respiratory diseases whereas a null NDVI-respiratory mortality relationship was uncovered based on a Korean population [16]. This discrepancy could be resulted from the un-measured confounders such as bacterial infection that may vary monthly or seasonally. Since our study focused on monthly NDVI exposure and adjusted for monthly time trend using spline function in the analysis, then we were more likely to minimize the bias due to bacterial infection compared with Kim’s findings that did not account for factors varied by month (e.g., infection rate of bacteria). It suggests controlling for time trend in the association between short-term NDVI exposure and respiratory mortality may be important to minimize confounding bias.

In addition to the evidence accumulating in Asian populations, findings uncovered in Caucasian populations indicate the potential benefit of greenness exposure on reducing mortality. A cross-sectional study based on aggregated information (i.e., lower level super output areas, LSOA) on greenness and mortality in the U.K. found the association of income deprivation with all-cause and circulatory disease mortality varied significantly across different exposures to green space, suggesting green environments may alleviate the health inequality related to income deprivation [7]. Another cross-sectional study based on individual profiles demonstrated variation of NDVI (tertile 3 vs. tertile 1) was associated with a 37% lower risk of hospitalization for cardiovascular diseases [8]. More recent cohort studies conducted in the U.S., Canada, and E.U. also had similar findings [6,9,10,11,12,13]. James et al. utilized the U.S.-based Nurses’ Health Study and revealed an 0.1 unit increment on cumulative average NDVI (250-m buffer) was associated with a 12% decrease on all-cause mortality [9]. Their cause-specific analysis on mortality found a stronger association between NDVI exposure for respiratory or kidney mortality compared with other causes of death (e.g., coronary heart diseases, stroke). The more prominent NDVI-mortality relationship on respiratory diseases (compared with CVD) was consistent with the findings uncovered in this study. Another two studies based on Canadian and Switzerland cohorts pointed to an association of greenness exposure with reduced all-cause mortality [11,12]. Additionally, these two studies found a stronger protective effect of NDVI exposure on respiratory mortality compared with death due to CVD. Orioli et al. conducted a study based on an Italian cohort, and their findings demonstrated an increment on greenness exposure (i.e., NDVI, leaf area index [LAI]) was associated with a 1.3% (NDVI) and 1.2% (LAI) decreased non-accidental mortality. A further mediation analysis showed the relationship between NDVI exposure and non-accidental mortality was mediated by PM_2.5_, NO_2_, or noise exposure given the proportion of mediation ranging from 27 to 92%. We observed an insignificant association between exposure to NDVI and lung cancer mortality, although a negative directionality was suggested. This null association could be resulted from the adjustment for PM_2.5_, a known environmental risk factor for lung cancer. Given the fact that greenness may reduce ambient PM_2.5_ levels, therefore adjustment for PM_2.5_ could attenuate the association of NDVI and lung cancer mortality. Several studies conducted in the U.K. and U.S. also found null association of NDVI with lung cancer mortality [7,18,19,20,21]. For example, Richardson et al. utilized an ecological design in the U.S. that covered 43 millions of city residents to examine relationship between greenness and lung cancer mortality, and findings showed no such relationship [21]. A recent case-control study conducted in Shanghai, China uncovered a null association between NDVI and lung cancer incidence [22].

Several mechanisms were proposed to support the protective effects of greenness exposure on reducing mortality. Dadvand et al. found schools surrounded by higher levels of greenness (i.e., NDVI) have lower levels of traffic-related air pollutants in Barcelona (Spain) [23]. An increment of NDVI within school was associated with 4.2 µg/m^3^ and 1.5 µg/m^3^ decreases in indoor NO_2_ and traffic-related PM_2.5_, respectively. The mitigation of air pollutants was more promising for schools with higher exposure to surrounding greenness compared with greenness within schools. A modeling study based on hourly measurement of air pollution in the U.S. suggested green vegetation in urban areas removed 711,000 metric tons of air pollutants (i.e., PM_10_, NO_2_, SO_2_, ozone, CO) [24]. In addition to the role of air pollution on greenness-mortality association, a study based on a questionnaire survey in Italy and the U.K. suggested green spaces might reduce heat stress [25]. Noise might be another factor linking greenness exposure and reduced mortality. Gidlof-Gunnarsson et al. found urban residents who had better availability to nearby green space have reduced noise annoyances and stress that might increase the risk of deaths [26].

Evidence from field experiments on human subjects in Japan and China showed exposure to greenness (e.g., forest bathing) may improve short-term physiological and psychological health [27,28,29,30,31,32]. Mao et al. recruited 20 healthy volunteers to evaluate the short-term effect of forest bathing on human health. They found participants exposed to short-term forest bathing had lower levels of pro-inflammatory responses (i.e., IL-6, TNF-α), stress markers (i.e., serum cortisol), and of mood state profiles (POMS) [31]. A similar finding was supported by another field experiment in Japan that demonstrated exposure to Shinrin-yoku (talking in the forest atmosphere or forest bathing) was associated with lower levels of cortisol, blood pressure, and pulse rate [28].

Several limitations should be recognized to correctly interpret the findings uncovered in this study. First, the nature of ecological research inherently has the limitation of ecological fallacy. Although several known factors (e.g., age structure, sex ratio, annual household income) contributing to mortality were controlled in the analyses, other potential confounders including smoking, alcohol consumption, or physical activity could not be included due to the lack of information at the township level. Second, we assessed the association of seasonal NDVI with mortality where a short-term effect of greenness was assumed. Most observations studies based on cohort profiles support the long-term effect of NDVI on mortality, and therefore more studies are needed to confirm our findings. However, a recent study in China found contemporaneous exposure NDVI was associated with reduced all-cause mortality [15], suggesting the potential short-term health benefit of greenness exposure. Third, we utilized the greenness exposure at the township levels instead of a more precise geographical unit (e.g., villages or census-blocks) because the place of death provided by the Taiwan Death Certificate Database was mosaic to township levels due to confidentiality issues. We potentially introduced certain levels of exposure misclassification that usually bias the association toward the null if a non-differential misclassification setting existed. Our study had several strengths, including utilizing a national representative dataset (i.e., Taiwan Death Certificate Database), controlling for air pollution, and a relative long-term observation from 2006–2014.

## 5. Conclusions

In sum, we found exposure to greenness was associated with reduced all-cause and cause-specific mortality in an Asian country. To reduce deaths from major diseases, we suggest government and related stakeholders increase green coverage at the community scale to improve general health quality. Given the inherent limitation of ecological design in this study, future studies may examine the protective effect of greenness on mortality based on individual information in a prospective design.

## Figures and Tables

**Table 1:** Demographic Information from 2006 to 2014.

Variables	Seasonal Mean (SD) ^a^
Number of Deaths per 100,000 people	
All Cause	96.09 (93.63)
Cardiovascular	21.91 (21.76)
Respiratory	9.42 (9.87)
Lung Cancer	5.89 (6.61)
Sex Ratio (Male/Female)	107.74 (8.11)
Income (thousands of NTD)	712.79 (166.03)
Age Structure (%)	
0–14	14.97 (3.07)
15–64	71.86 (2.97)
>64	13.17 (4.21)
Index of Greenness	
NDVI	0.51 (0.17)
Area of Forest (%)	30.1 (30.8)
Area of Park (%)	1.17 (2.99)
Air Pollutants (μg/m^3^)	
PM_2.5_	27.61 (11.27)
NO_X_	22.72 (8.62)
Precipitation (mm)	601.9 (530.78)
Temperature (°C)	22.5 (4.08)

^a^ The mean and standard deviation (SD) were based on the seasonal statistics for each variable.

**Table 2:** Association between NDVI and Mortality.

	All-Cause ^d^	Cardiovascular ^d^	Respiratory ^d^	Lung Cancer ^d^
Model 1 ^a^	0.901 ^¶^ (0.862, 0.941)	0.892 ^‡^ (0.817, 0.975)	0.721 ^¶^ (0.632, 0.824)	0.871 (0.735, 1.032)
Model 2 ^b^	0.896 ^¶^ (0.857, 0.936)	0.887 ^#^ (0.812, 0.97)	0.695 ^¶^ (0.608, 0.795)	0.866 (0.731, 1.026)
Model 3 ^c^	0.900 ^¶^ (0.86, 0.94)	0.892 ^‡^ (0.816, 0.975)	0.699 ^¶^ (0.611, 0.799)	0.884 (0.746, 1.048)

Abbreviations: NDVI, normalized difference vegetation index; RR: risk ratio; CI, confidence interval. ^a^ Model 1 was adjusted for total population, age, sex ratio, taxable income, precipitation, time trend, and temperature. ^b^ Model 2 was Model 1 with further adjustment for NO_x_. ^c^ Model 3 was Model 2 with further adjustment for urbanization levels. ^d^ Estimation of risk ratio (RR) and 95% confidence interval value was based on one-unit increment on NDVI. Significant of risk ratio (^‡^ *p* value < 0.05, ^#^ *p* value < 0.01, ^¶^ *p* value < 0.001).

**Table 3:** Stratified Analysis for NDVI-Mortality Association by Population Characteristics.

	All-Cause ^a^	Cardiovascular ^a^	Respiratory ^a^	Lung Cancer ^a^
Sex Ratio (Male/female)				
<107.3	0.877 ^¶^ (0.828, 0.928)	0.893 (0.797, 1.002)	0.759 ^#^ (0.640, 0.901)	0.843 (0.683, 1.040)
≥107.3	0.929 * (0.865, 0.997)	0.882 (0.767, 1.014)	0.683 ^¶^ (0.557, 0.836)	0.889 (0.682, 1.158)
Age 15–64				
<71.9%	0.924 ^‡^ (0.865, 0.987)	0.874 ^‡^ (0.767, 0.996)	0.661 ^¶^ (0.545, 0.801)	0.818 (0.638, 1.048)
≥71.9%	0.876 ^¶^ (0.824, 0.931)	0.892 (0.789, 1.008)	0.767 ^#^ (0.639, 0.921)	0.831 (0.662, 1.043)
Annual Household Income, NTD				
<668,701	0.954 (0.889, 1.025)	0.882 (0.766, 1.016)	0.739 (0.602, 0.908)	0.757 (0.581, 0.987)
≥668,701	0.871 (0.822, 0.922)	0.881 (0.785, 0.988)	0.733 (0.618, 0.871)	0.899 (0.729, 1.108)
Area				
Rural	0.923 (0.867, 0.982)	0.863 (0.763, 0.976)	0.733 (0.612, 0.879)	0.83 (0.651, 1.057)
Urban	0.861 (0.806, 0.919)	0.903 (0.791, 1.030)	0.72 (0.590, 0.879)	0.981 (0.771, 1.247)

Abbreviations: NDVI, normalized difference vegetation index; RR: risk ratio; CI, confidence interval; NTD, New Taiwan Dollar. ^a^ All models were adjusted for total population, age, sex ratio, taxable income, precipitation, time trend, and temperature. Estimation of risk ratio (RR) was based on one unit increment on NDVI. Significant of risk ratio (^‡^ *p* value < 0.05, ^#^ *p* value < 0.01, ^¶^ *p* value < 0.001).

**Table 4:** Relationship between Different Green Index and Mortality.

Greenness Index	All-Cause ^a^	Cardiovascular ^a^	Respiratory ^a^	Lung Cancer ^a^
NDVI	0.901 ^¶^ (0.862, 0.941)	0.892 ^‡^ (0.817, 0.975)	0.721 ^¶^ (0.632, 0.824)	0.871 (0.735, 1.032)
Forest Area (%)	0.883 ^¶^ (0.866, 0.900)	0.898 ^¶^ (0.880, 0.917)	0.903 ^¶^ (0.883, 0.923)	0.884 ^¶^ (0.864, 0.904)
Forest or Park Area (%)	0.884 ^¶^ (0.867, 0.901)	0.898 ^¶^ (0.88, 0.917)	0.903 ^¶^ (0.883, 0.923)	0.885 ^¶^ (0.865, 0.905)

Abbreviations: NDVI, normalized difference vegetation index; RR: risk ratio; CI, confidence interval. ^a^ All models were adjusted for total population, age, sex ratio, taxable income, precipitation, time trend, and temperature. Estimation of risk ratio (RR) was based on one unit increment on NDVI and 10% increment on area of the forest/park. Significant of risk ratio (^‡^ *p* value < 0.05, ^¶^ *p* value < 0.001).
